# National-sectoral emission constraints in PyPSA-based open-source European energy system models

**DOI:** 10.1016/j.mex.2023.102014

**Published:** 2023-01-14

**Authors:** Leon Joachim Schwenk-Nebbe

**Affiliations:** a Department of Mechanical and Production Engineering, Aarhus University, Denmark; b iCLIMATE Interdisciplinary Centre for Climate Change, Aarhus University, Denmark; c Centrica Energy Trading A/S, Ørestads Boulevard 73, 9th floor, København S 2300, Denmark

**Keywords:** Open-source energy modelling, Sector coupling, Emission reductions, European energy system and policy, National-sectoral PyPSA-Eur-Sec emission handling

## Abstract

The proposed sectorial and national-sectorial emissions accounting methods for the PyPSA-Eur-Sec model adds a layer of abstraction to the model that allows decarbonisation at defined rates of individual sectors. PyPSA-Eur-Sec is a sector-coupled energy model of the European energy system and includes the electricity, heating, transportation, and industry sectors. The model and this extension are fully open-source, and all data sources and cost assumptions are openly available. The model allows for transparent, reliable, and computationally efficient analyses. These can form a solid basis for energy investments and policy advice. Additionally, for the first time, we present a diagram of the inner workings of the PyPSA-Eur-Sec model. We visualise exactly the possible energy flows, energy conversions, and couplings between the sectors in the model.

• Future cost-optimal European energy system configurations can be obtained from the PyPSA-Eur-Sec model under a given carbon dioxide emission budget.
• This model extension allows steering the emissions individually in the four modelled sectors electricity, heating, transportation, and industry.
• We provide a visual overview of the energy flows and conversions optimised in the model.

## Graphical abstract

**Figure:**
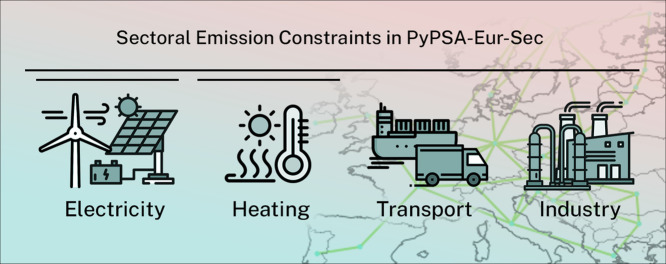

Specifications Table

**Table:** 

Subject Area:	Energy
More specific subject area:	Energy system modelling
Method name:	National-sectoral PyPSA-Eur-Sec emission handling
Name and reference of original method:	PyPSA-Eur-Sec: T. Brown, M. Victoria, L Zeyen, F. Neumann, L. Schwenk-Nebbe, & M. Frysztacki. (2021). PyPSA/pypsa-eur-sec: PyPSA-Eur-Sec Version 0.5.0 (v0.5.0). Zenodo. 10.5281/zenodo.4778332
Resource availability:	The proposed model adaption is open-source and available at: L.J. Schwenk-Nebbe. (2021). PyPSA-Eur-Sec-Sectoral-Emissions (v0.5.0). Zenodo. 10.5281/zenodo.5718590

*Method details

Sectoral and national-sectoral emissions reduction caps enable the investigation of specific emission goals in the individual sectors. The purpose of the method is to allow the study and comparison of energy system configurations in which different sectoral emission reduction goals are fulfilled. The overall purpose is to enable the development of open, trusted, and nuanced policy advice.

## Motivation for the proposed adaptation

The original method includes the possibility of setting a global CO_2_ emission cap, thereby allowing the simulation and assessment of highly renewable future energy systems. Having a single emission cap, the decarbonisation pressure forces the cheapest-to-transit technologies and sectors to become renewable first. Even though this might at first seem economically desired, several potentially undesired effects can occur, and no control of where decarbonisation efforts are applied is given. In a pure cost-optimisation, the sectors are decarbonised one by one instead of simultaneously. This leads to rapid deployments of capacity that might lead to the rise and quick fall of investment opportunities leading to unstable business incentives. The proposed method allows for detailed studies of varying sectoral emissions and thereby provides a possibility to gain a deeper understanding of the issues as well as giving the modeller control over the individual sectoral emissions.

## Technical details of the model

The model is based on the open-source model PyPSA-Eur-Sec, first introduced in [2], here applied as version 0.5.0 [1], and actively developed on GitHub [3]. The model is based on the energy system optimisation framework Python for Power System Analysis [4] (PyPSA) (version 0.17.1). The main dependency of PyPSA-Eur-Sec is on the projects PyPSA-Eur [5] (version 0.3.0) as the underlying electricity model, atlite [6] (version 0.0.2) for renewable generation potentials, and the Technology Data (version 0.2.0) for technology efficiency, lifetime, and cost data. The three projects are likewise open-source and distributed on the same GitHub channel.

In the PyPSA model, the system configuration is optimised according to the following minimisation problem that is evaluated for every bus n, technolody s, and timestep t:

(1) $$\min_{G_{n,s},E_{n,s},F_{l},g_{n,s}\left(t\right)}\left[\sum_{n,s}c_{n,s}\cdot G_{n,s}+\sum_{n,s}{\hat{c}}_{n,s}\cdot E_{n,s}+\sum_{l}c_{l}\cdot F_{l}+\sum_{n,s,t}o_{n,s,t}\cdot g_{n,s,t}\right].\;$$

**Fig. 1:**
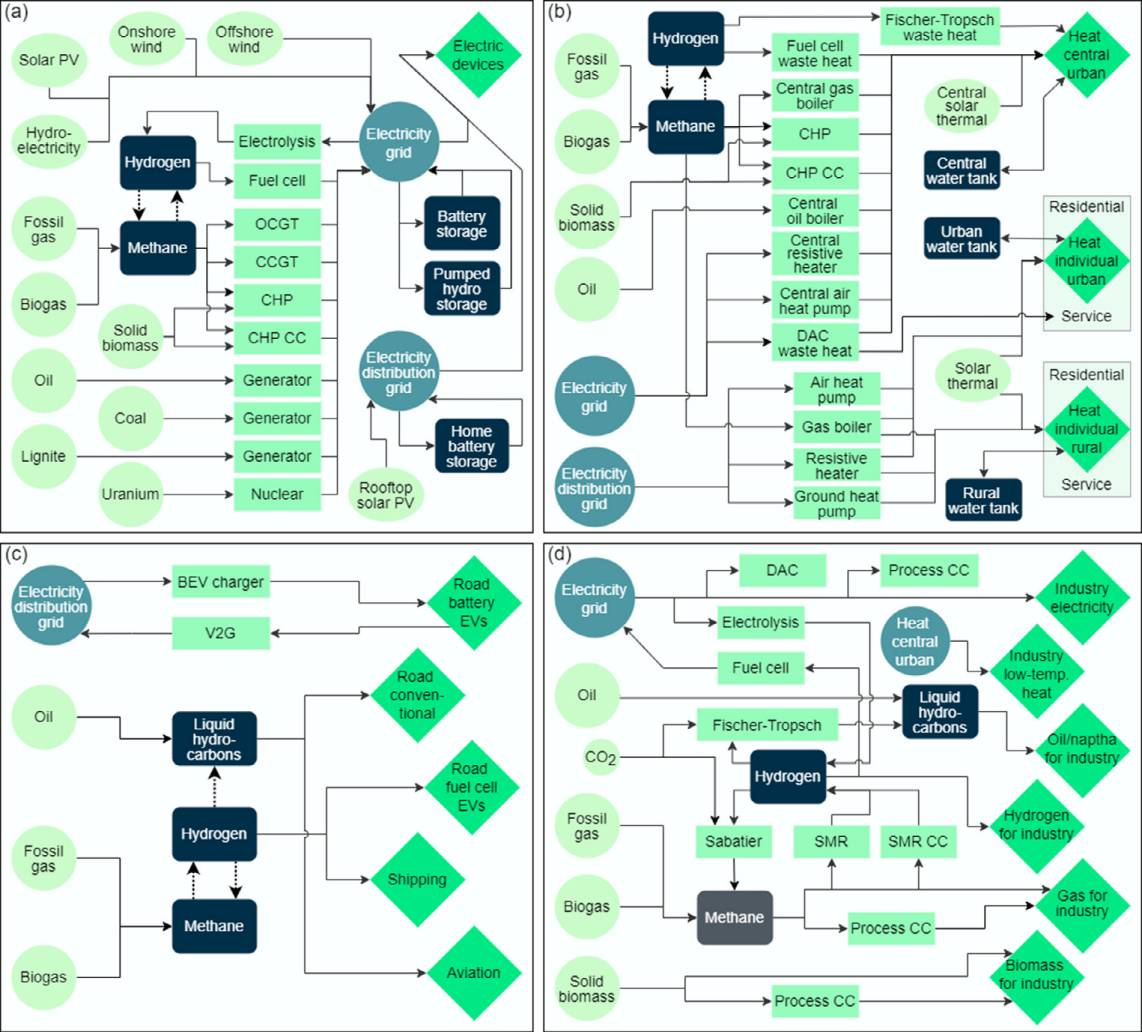
Showing a diagram of the specific implementation of the PyPSA-based model of the European energy system applied in this study. The model visualisation is split into the four modelled sectors that are shown in the respective panels. Panel (a) shows the electricity sector, Panel (b) the heating sector, Panel (c) the transportation sector, and Panel (d) the industry and conversion sector. Energy originates from variable renewable generators (ovals) and energy carriers (circles) and is converted (rectangles) to supply loads (diamonds). Note that the conversion between hydrogen and methane is shown in full detail only in Panel (d) while it is simplified in the remaining panels. In Panel (b), note that the individual urban and individual rural heating demand is split into two time series, respectively, for the residential and services customers. In Panel (d), note that the low-temperature industrial heat demand is supplied in the same way as the urban central heat demand in the heating sector. For simplicity, the diagrams only show which processes require CO_2_ and not its sources. The utilised CO_2_ is obtained from carbon-capture (CC) and direct-air-capture (DAC).

Here, $c_{n,s}$ denote the fixed annualised investment costs of the generator and storage power capacities $G_{n,s}$, while ${\hat{c}}_{n,s}$ denote the fixed annualised costs of the storage energy capacities $E_{n,s}$, $c_{l}$ denote the fixed annualised costs of the bus connectors $F_{l}$, and lastly $o_{n,s,t}$ denote the variable costs of generator and storage dispatches $g_{n,s,t}$. Bus connectors l include AC and DC transmission lines but also converters between different buses – see Fig. 1 below for the details. The optimisation objective is hence the total annualised system cost that consists of capital investment and variable operational costs. The optimisation is subject to several constraints like the nodal energy balance ensuring that all energy needs are fulfilled at every node and timestep and the global CO_2_ constraint ensuring that the combined emissions remain below a given threshold. For the full implementation details see [1], [2], [3]. The global emission constraint is introduced directly into the minimisation problem as inequality constraints limiting the CO_2_ emissions to an upper limit $CAP_{CO_{2},global}$:

(2) $$\sum_{n,s,t}\varepsilon_{s}\frac{g_{n,s,t}}{\eta_{n,s}}+\sum_{n,s}\varepsilon_{s}\left(e_{n,s,t=0}-e_{n,s,t=T}\right)\leq CAP_{CO_{2},global}\;↔\;\mu_{CO_{2},global},\;$$

where $\varepsilon_{s}$ denote the CO_2_-tonne-per-MWh_th_ emissions of the fuels s, $\eta_{n,s}$ denote the generator efficiencies, and $e_{n,s,t}$ denote the state of charge of the storages which include CO_2_ sequestration. The Lagrange multiplier $\mu_{CO_{2},global}$ represents the global shadow price of CO_2_. It is interpreted as the price per tonne of CO_2_ that emitting producers need to pay so that the price competition on the open market between emitting and non-emitting producers results in staying below the set emission cap. If the emission cap $CAP_{CO_{2},global}$ is set large enough the constraint is non-binding, and the resulting CO_2_ price is zero.

We introduce additional constraints to limit the emissions from individual sectors and/or countries. They are similarly introduced directly into the minimisation problem as inequality constraints limiting the CO_2_ emissions by emitters attributed to sectors and countries to individual upper limits $CAP_{CO_{2},\left\{n,s\right\}}$:

(3) $$\sum_{t,\left\{n,s\right\}}\varepsilon_{s}\frac{g_{n,s,t}}{\eta_{n,s}}+\sum_{\left\{n,s\right\}}\varepsilon_{s}\left(e_{n,s,t=0}-e_{n,s,t=T}\right)\leq CAP_{CO_{2},\left\{n,s\right\}}\;↔\;\mu_{CO_{2},\left\{n,s\right\}}\;\forall \;CAP_{CO_{2},\left\{n,s\right\}}\;.$$

The extended model allows to set a global emission constraint, national emission constraints, individual emission targets for the four sectors, or national-sectoral emission constraints that set upper emission caps on both national and sectoral levels. In the case of national-sectoral emission caps, one constraint per country and sector pair is implemented. Subsequently, the realised national-sectoral CO_2_ emissions are determined from the optimised dispatch. The national-sectoral CO_2_ prices are again determined as the Lagrange multipliers (dual variables) from their respective constraints. Hence, the emission prices are an output from the model.

The more emission constraints are implemented the larger the chance that they overlap and that some become non-binding. If multiple emission constraints are overlapping, like in the case of having both a constraint on the power sector and a global constraint on the whole energy system, the interpretation of the CO_2_ prices changes. A generator that is subject to multiple emission constraints will pay only the largest of the respective CO_2_ prices.

## Sectoral emission constraints and implementation details

We apply the sectoral emissions constraints on the direct sectoral emissions instead of the indirect emissions. This allows us to detangle the interconnected sectors. Subsequently, the indirect sectoral emissions can be obtained from the optimised model. All CO_2_ emission reductions are given in per cent relative to their corresponding 1990-levels.

The emissions in the transportation sector are exogenously defined by a fuel-switching parameter for road transport. This parameter sets the share of internal combustion engine (ICE) vehicles, battery electric vehicles (BEVs), and fuel cell electric vehicles (FCEVs). Half of the BEVs are assumed to do smart charging and allow feed-in back to the grid. Shipping demand is assumed to be supplied by hydrogen and aviation demand via traditional oil-based fuels. Thereby, the transport sector emissions are defined fully exogenously, and no additional sectorial constraint is necessary.

The electricity sector and heating sector share the emissions from combined heat and power (CHP) units proportional to the respective electricity and heat output. For both sectors, direct emission constraints are implemented following Eq. (3). In the case of national-sectoral emission caps, one constraint per country is necessary for both sectors.

The remaining emissions are attributed to the industry sector and limited by the global emission constraint. Hence, no additional sectoral constraint is necessary. For steelmaking in the industry sector, we presume the use of Direct Reduced Iron (DRI) in the Electric Arc Furnace (EAF). In the model, we assume an exogenous fuel switching for the production of steel where we set the fraction of steel produced via the primary route (DRI + EAF) against the secondary route (EAF). Similarly, we assume for the production of aluminium a fraction via the primary route versus scrap reuse. We disable the possibility of CO_2_ sequestration in the model to make sure that no carbon leakage between the sectors occurs.

The sectoral and national-sectoral emission caps are set directly in the configuration file (*config.yaml*), while the constraint definition is included in the script files *scripts/prepare_sector_network.py* and *scripts/solve_network.py*.

## Energy flows and conversions

The following figure shows a diagram overview of the main components and energy flows in the applied model divided into the four energy sectors entailed.

The heating demand is split into five individual load time series since both the individual rural and urban heating branches have residential and services consumers, respectively. In both cases, the residential and services branches can be supplied by the same technologies apart from the individual urban branch where the services demands can additionally be supplied by waste heat from DAC. Negative emission technologies (like carbon capture) are assumed to be part of the industry sector. The generation of synthetic electrofuels is likewise included in the industry sector.

## Data foundation and assumptions

**Fig. 2:**
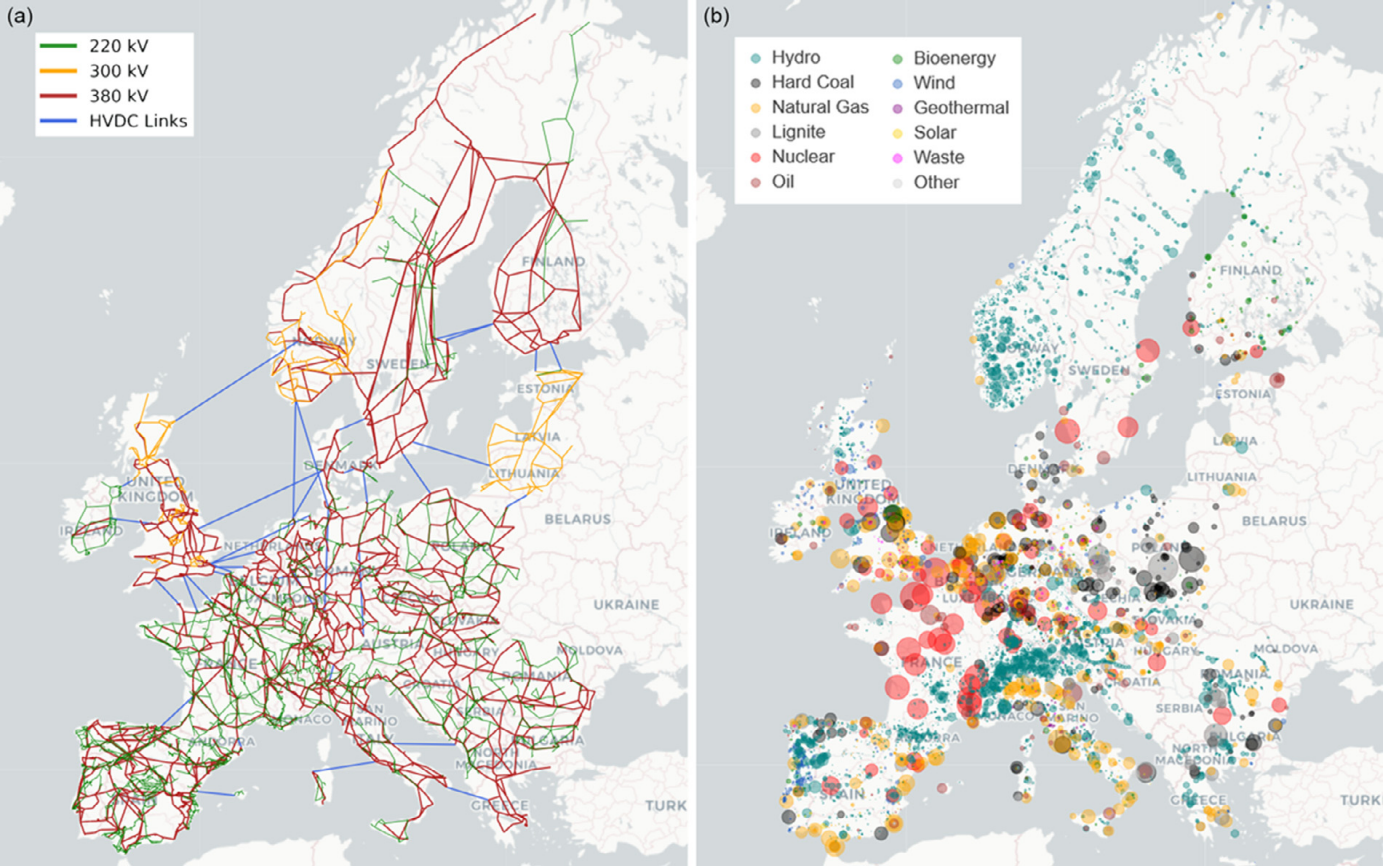
Showing the basis of the power sector description in the model. Panel (a) shows the full detail of the cleaned and processed network topology which is clustered to a given number of nodes in the model and approximated by a single voltage level to increase the optimisation speed. Panel (b) gives an overview of all the existing power plant capacities that form the brownfield description in the model. The size of the disks corresponds to the capacities of the plants. As a reference note that the Forsmark nuclear reactor north of Stockholm in Sweden has a nameplate capacity of 3291 MW.

The model performs a brownfield optimisation, i.e. taking today's capacities as a starting point for the further build out of the European energy system. It includes a detailed description of the European transmission network as shown in Fig. 2 (a). In order to solve the model in reasonable time, the network topology is clustered down to a lower degree of nodes and interconnections. The system is modelled as a brownfield optimisation. It takes today's existing capacities as a starting point if they are still assumed to be operational in the model year based on their technology lifetime. Hence, depending on the set model year not all the shown power plants from Panel (b) in Fig. 2 will be part of the model. Furthermore, the political decision of an earlier-than-technically-expected nuclear phase-out in the countries Italy, Belgium, Germany, Spain, and Switzerland before 2030 is reflected in the model. In these countries the existing brownfield nuclear capacities are removed but the optimiser could still install new nuclear capacity as in the other countries.

**Fig. 3:**
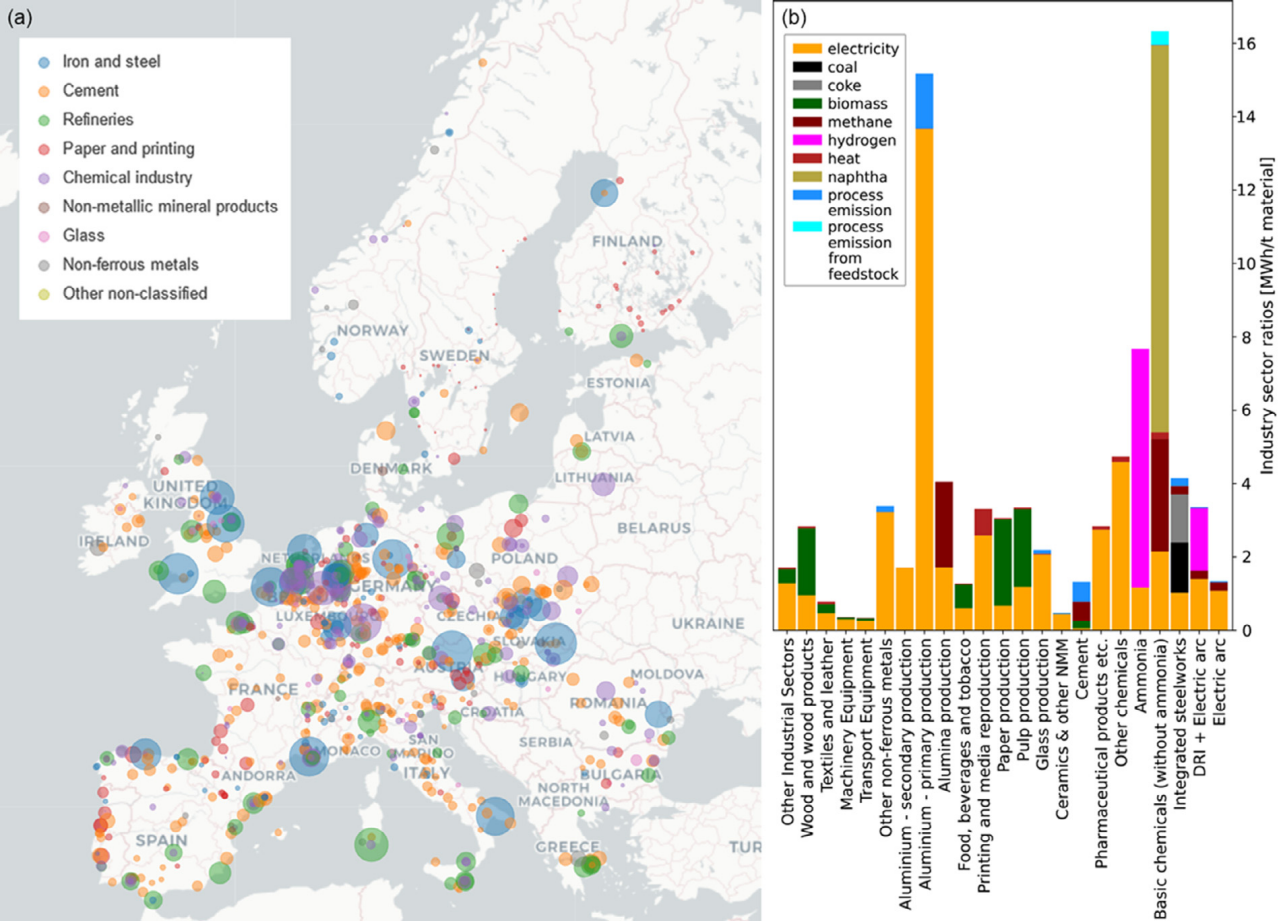
Industry sector modelling choices. Panel (a) shows the industrial sites that form the basis of the geographic distribution of industry sector demands in the model. The colour corresponds to the indicated industrial sectors and the size of the disks indicates the annual CO_2_ emissions. As a reference the industrial cement production site Aalborg Portland A/S in northern Denmark emits 1.72 GtCO_2_/a. Panel (b) shows key industry ratios that indicate which fuels fulfil which industrial demands and how energy intensive the processes are. NMM denotes non-metallic minerals and DRI denotes Direct Reduced Iron.

On Fig. 3 key industry sector model assumptions are shown. Note the large difference in energy demand between the primary and secondary route for aluminium production. Similarly, for steel production the primary route of Direct Reduced Iron (DRI) in the Electric Arc Furnace (EAF) using hydrogen is significantly more energy intensive than the secondary route. In the model, it is assumed that the current industrial demand remains constant and that the locations of the industrial demand sites remain the same. This is a strong assumption but an adequate estimate for a near-future.

## Comparison to other software

PyPSA combines the strengths from traditional power system models with the universality from energy system models. Power system models offer their detailed electrical methodology that includes the physics of power flows while energy system models in turn include investment optimisation and energy system coupling. Hence, PyPSA can be applied for both purposes, but its real strength lies in combining the two approaches. PyPSA is often compared to the power system model PYPOWER [8] and the energy system models Calliope [9], oemof [10], OSeMOSYS [11]. These models offer no pre-configured implementation of sectoral emission constraints, but user-defined custom constraints are supported and could be utilised to implement such features in an analogous way as described in this publication. All optimization tools can in general be used to model sectoral emission constraints, which are limits on the amount of greenhouse gas emissions that can be produced by a particular sector of the energy system. In PYPOWER, sectoral emission constraints can be implemented using the user constraint function (UCF) feature. UCFs allow users to specify custom constraints on the optimization problem, and can be used to implement sectoral emission constraints by limiting the amount of emissions produced by each generator in the system. In Calliope, sectoral emission constraints can be implemented using the "emission_limit" parameter in the "tech" module. In oemof, sectoral emission constraints can be implemented using the "emission_limit" parameter in the "Transformer" component. In OSeMOSYS, sectoral emission constraints can be implemented using the "MAX_EMIS" parameter in the "emission" module. In the PLEXOS software [12], sectoral emission constraints can be implemented using the "Emission Limit" parameter in the "Commitment" module. These parameters can be set to specify a maximum amount of emissions that can be produced by a particular generator. Note that the specific implementation of sectoral emission constraints may vary somewhat between the different tools we just give a general overview of how such efforts should be realised. Review the documentation for the specific tool to determine the best way to implement sectoral emission constraints in each model. The shortcoming of these manual workarounds is that the user would need to create separate emission types and constraints for each sector or region, which may require more effort and may be more difficult to understand and manage. Nevertheless, PyPSA offers a fully sector coupled energy system model that includes more detailed modelling of power networks than its competitors and includes CO_2_ flows. Likewise, the PyPSA software architecture was designed around emission constraints and thereby integrates these constraints directly instead of having to build a custom module around it. The software that comes closes to PyPSA in terms of functionality is PLEXOS in which it is also possible to set emissions caps and allocate allowances. However, this framework is neither free nor open source and the inner workings can thereby not be compared. For a detailed comparison of PyPSA to other available software see Table 3 in [4].

## Additional information

Please note that the PyPSA-Eur-Sec model is still under active development. Extensive changes, as well as vital additions to the model, are still occurring frequently. Changes to the methodology are likewise possible to occur. Please see [3] for the current state of the development and the discussion of proposed changes. The adapted method is available in [7].

## Declaration of Competing Interest

The authors declare that they have no known competing financial interests or personal relationships that could have appeared to influence the work reported in this paper.

## Data Availability

Data will be made available on request.

## References

[1] T. Brown, M. Victoria, L. Zeyen, F. Neumann, L. Schwenk-Nebbe, & M. Frysztacki. (2021). PyPSA/pypsa-eur-sec: PyPSA-Eur-Sec version 0.5.0 (v0.5.0). Zenodo. doi:10.5281/zenodo.4778332.

[2] Brown T., Schlachtberger D., Kies A., Schramm S., Greiner M. (2018). Synergies of sector coupling and transmission reinforcement in a cost-optimised, highly renewable European energy system. Energy.

[3] T. Brown, F. Neumann, M. Victoria, L. Zeyen, L. Schwenk-Nebbe, M. Frysztacki, & F. Witte. (2021). PyPSA/pypsa-eur-sec. GitHub. https://github.com/PyPSA/pypsa-eur-sec.

[4] Brown T., Hörsch J., Schlachtberger D. (2018). pypsa: python for power system analysis. J. Open Res. Softw..

[5] Hörsch J., Hofmann F., Schlachtberger D., Brown T. (2018). PyPSA-Eur: an open optimisation model of the European transmission system. Energy Strat. Rev..

[6] Hofmann F., Hampp J., Neumann F., Brown T., Hörsch J. (2021). atlite: a lightweight python package for calculating renewable power potentials and time series. J. Open Source Softw..

[7] L.J. Schwenk-Nebbe. (2021) PyPSA-Eur-Sec-Sectoral-emissions (v0.5.0). Zenodo. doi:10.5281/zenodo.5718590.

[8] R. Lincoln. (2017) PYPOWER https://github.com/rwl/PYPOWER.

[9] Pfenninger S. (2017). Dealing with multiple decades of hourly wind and PV time series in energy models: a comparison of methods to reduce time resolution and the planning implications of inter-annual variability. Appl. Energy.

[10] S. Hilpert, S. Günther, C. Kaldemeyer, U. Krien, G. Plessmann, F. Wiese, and C. Wingenbach. (2017) Addressing energy system modelling challenges: the contribution of the open energy modelling framework (oemof). Preprints. doi:10.20944/preprints201702.0055.v1.

[11] Howells M., Rogner H., Strachan N., Heaps C., Huntington H., Kypreos S., Hughes A., Silveira S., DeCarolis J., Bazillian M., Roehrl A. (2011). Osemosys: the open source energy modeling system: an introduction to its ethos, structure and development. Energy Policy, Sustain. Biofuels.

[12] Energy Exemplar. (2017) PLEXOS http://energyexemplar.com/.

